# A functional assay to classify *ZBTB24* missense variants of unknown significance

**DOI:** 10.1002/humu.23786

**Published:** 2019-06-18

**Authors:** Haoyu Wu, Kelly K. D. Vonk, Silvère M. van der Maarel, Gijs W.E. Santen, Lucia Daxinger

**Affiliations:** ^1^ Department of Human Genetics Leiden University Medical Centre Leiden The Netherlands; ^2^ Department of Clinical Genetics Leiden University Medical Centre Leiden The Netherlands

**Keywords:** CAKUT, ICF syndrome, VUS, ZBTB24

## Abstract

Increasing use of next‐generation sequencing technologies in clinical diagnostics allows large‐scale discovery of genetic variants, but also results in frequent identification of variants of unknown significance (VUSs). Their classification into disease‐causing and neutral variants is often hampered by the absence of robust functional tests. Here, we demonstrate that a luciferase reporter assay, in combination with ChIP‐qPCR, reliably separates pathogenic *ZBTB24* missense variants in the context of immunodeficiency, centromeric instability, facial anomalies (ICF) syndrome from natural variants in healthy individuals and patients of other diseases. Application of our assay to two published *ZBTB24* missense VUSs indicates that they are likely not to cause ICF2 syndrome. Furthermore, we show that rare gnomAD *ZBTB24* missense variants in key residues of the C2H2‐ZF domain lead to a loss of function phenotype that resembles ICF2, suggesting that these individuals are carriers of ICF syndrome. In summary, we have developed a robust functional test to validate missense variants in *ZBTB24*.

Genome‐wide sequencing has now become routine in many diagnostic laboratories and identifies many genetic variants per individual (Gonzaga‐Jauregui, Lupski, & the Gibbs, 2012; Peterson, Doughty, & Kann, 2013). Therefore, separating disease‐causing from neutral variants is crucial. Depending on the disease and the specific inheritance pattern, segregation studies may be an efficient way of reducing the number of potentially pathogenic variants. Another important resource in the interpretation of variants comes from large databases (Peterson et al., 2013). For example, the Genome Aggregation Database (gnomAD) (http://gnomad.broadinstitute.org/; Lek et al., 2016) provides information about variants from a total of 141,456 unrelated individuals, which is depleted of early‐onset diseases. There are also databases for potentially pathogenic variants, such as ClinVar (https://www.ncbi.nlm.nih.gov/clinvar/; Landrum et al., 2016) and HGMD (https://www.hgmd.cf.ac.uk/ac/index.php; Stenson et al., 2003). Nonetheless, a large number of variants cannot be reliably classified and they remain variants of unknown significance (VUSs). In particular missense variants and small in‐frame insertions/deletions in exons or introns (Abecasis et al., 2010) are difficult to interpret, especially for recessive disorders since segregation analysis is not helpful. Functional studies demonstrating the effects of VUS on a biological function can provide important clues to draw a conclusion whether a VUS is disease‐associated or not (Woods et al., 2016).

Pathogenic variants in the zinc finger and BTB domain containing 24 genes (*ZBTB24*) cause immunodeficiency, centromeric instability, facial anomalies (ICF) syndrome type 2 (ICF; MIM# 242860, 614069; de Greef et al., 2011). ICF is a life‐threatening, genetically heterogeneous, autosomal‐recessive disorder characterized by hypo‐ or agammaglobulinemia and DNA hypomethylation of repetitive DNA. Pathogenic variants in four genes have been shown to cause ICF syndrome: *DNMT3B* (ICF1), *ZBTB24* (ICF2), *CDCA7* (ICF3), and *HELLS* (ICF4) (de Greef et al., 2011; Thijssen et al., 2015; Xu et al., 1999). ZBTB24 belongs to the zinc finger and BTB domain containing (ZBTB) family, and pathogenic variants in *ZBTB24* account for about 30% of ICF cases (de Greef et al., 2011; Weemaes et al., 2013). ZBTB24 contains N‐terminal BTB and AT‐hook domains, and eight C‐terminal C2H2 zinc finger (C2H2‐ZF) domains. BTB domains (broad‐complex, tramtrack, and bric‐a‐brac) are present in many species and involved in protein‐protein interactions including homo‐ and heterodimerization (Bonchuk, Denisov, Georgiev, & Maksimenko, 2011; Stogios, Downs, Jauhal, Nandra, & Prive, 2005). An AT‐hook domain is a DNA binding motif that has been reported to interact with AT‐rich DNA sequences (Lyst, Connelly, Merusi, & Bird, 2016). C2H2‐ZF domains are well‐studied and responsible for DNA binding. The double cysteines and histidines are highly conserved in the C2H2‐ZF protein family, though the sequences in between are highly variable, indicating that the C2H2 array could be crucial for the structure of the zinc finger (Najafabadi et al., 2015; Wolfe, Nekludova, & Pabo, 2000).

To date, 13 homozygous *ZBTB24* nonsense variants have been identified from ICF2 patients, with seven variants located in the C2H2‐ZF domains, two variants in the BTB domain, one in the AT‐hook domain and three in between the AT‐hook and C2H2‐ZF domains (Figure 1a; Chouery et al., 2012; Conrad, Dawany, Sullivan, Devoto, & Kelsen, 2017; de Greef et al., 2011; Kamae et al., 2018; Nitta et al., 2013; van den Boogaard et al., 2017; Weemaes et al., 2013). These nonsense variants are predicted to result in proteins with a premature stop codon (Figure 1a). In addition, two homozygous *ZBTB24* missense variants located in the C2H2‐ZF have been identified in ICF2 patients (Cerbone et al., 2012; Nitta et al., 2013; Figure 1a), suggesting a crucial role for the C2H2‐ZF in ZBTB24 function.

**Figure 1:**
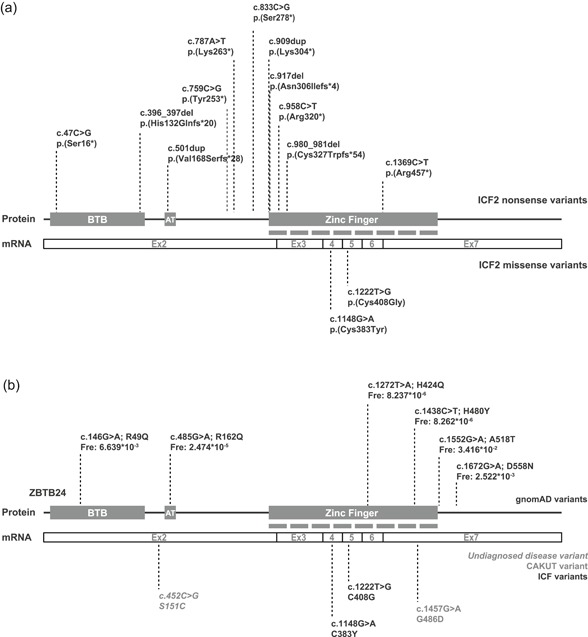
Variants in *ZBTB24.* (a) Schematic of ZBTB24 mRNA and protein. ZBTB24 contains a BTB and AT‐hook domain located in exon 2, and 8 C2H2 zinc finger domains that span from exon 2 to 7. *ZBTB24*/ICF2 nonsense variants and (bottom) missense variants with cDNA and amino acid changes. (b) Schematic of ZBTB24 mRNA and protein. gnomAD variants (black) including cDNA and amino acid changes, as well as allele frequencies, are shown. Fre: allele frequency. (bottom) Variants from undiagnosed disease network (gray and italic), CAKUT (gray) and ICF2 patients (black) with cDNA and amino acid changes. CAKUT, congenital anomalies of the kidneys and urinary tract; ICF, immunodeficiency, centromeric instability, facial anomalies

Recently, two *ZBTB24* missense VUSs have been identified, which, based on the related clinical phenotypes, are suspected to be damaging. In a patient with congenital anomalies of the kidneys and urinary tract (CAKUT; MIM# 610805, 143400, 618270), a homozygous missense variant was found in the C2H2‐ZF domain (Vivante et al., 2017; Figure 1b). Furthermore, the Undiagnosed Diseases Network (UDN; https://undiagnosed.hms.harvard.edu/) reported a single heterozygous VUS in *ZBTB24* in a 2‐year‐old girl diagnosed with primary immunodeficiency and an absent thyroid (Figure 1b). Finally, a gnomAD search revealed that numerous *ZBTB24* missense variants, including variants in the C2H2‐ZF domain, have been described in individuals without early‐onset disease. Yet, for all these cases, it remains unclear whether the *ZBTB24* variants indeed alter ZBTB24 function and are pathogenic. Here, we report a reliable method to distinguish pathogenic variants and VUS in *ZBTB24*.

The Ty1_*ZBTB24* (RefSeq NM_014797.2) vector was generated before (Wu et al., 2016). Site‐directed mutagenesis was used to introduce *ZBTB24* variants. In brief, primers containing different variants were designed and polymerase chain reaction (PCR) was performed with AmpliTaq Gold^TM^ DNA Polymerase (N8080241; Thermo) using 10 ng of template plasmid with the following program, 95°C for 5 min, (95°C for 30 s, 55°C for 30 s, 72°C for 10 min, repeat for 16 cycles), 72°C for 6 min. After amplification, PCR products were cooled down on the ice for 2 min, and digested with DpnI (R0176S; NEB) at 37°C for 1 hr to digest the template plasmids. Next, PCR products were transformed into competent *E. coli* (DH5α). In brief, cells and PCR product were incubated on ice for 30 min. Heat shock was performed at 42°C for 45 s and cells were immediately put back on ice for 2 min. Cells were allowed to recover at 37°C for 30 min, plated on LB plates with 0.1 mg/ml Kanamycin (10106801; Roche) and incubated overnight at 37°C. On the next day, colonies were picked for Miniprep. All vectors were sequenced to validate the introduced variants. Primers used for mutagenesis are shown in Table S1. Generation of vectors for the Luciferase reporter assay was described before (Wu et al., 2016).

Mouse embryonic stem cells (mESCs) were cultured in serum plus 2i condition (Knockout DMEM [10829‐018; Gibco], 10% FBS [DE14‐801F; BioWhittaker], NEAA [11140; Gibco], l‐glutamine [25030‐123; Gibco], sodium pyruvate [11360; Gibco], 2‐Mercaptoethanol [31350; Gibco] and leukemia inhibitory factor [ESG1107; Millipore] supplemented with MEK inhibitor PD0325901 [1 mM] and GSK3 inhibitor CHIR99021 [3 mM; Axon Medchem]) on 0.1% gelatin. U2OS cells were culture in DMEM (31966‐021; Gibco) supplemented with 10% FCS (10270‐106; Gibco) and 1% Pen‐Strep (15140‐122; Gibco). The cell lines tested negative for mycoplasma contamination on a regular basis.

U2OS cells were lysed in cell lysis buffer (20 mM triethanolamine [T1377; Sigma], 0.14 M NaCl, 0.1% Sodium deoxycholate [D6750; Sigma], 0.1% sodium dodecyl sulfate [SDS], 0.1% Triton X‐100) with protease and phosphatase inhibitor cocktail (04906837001; Roche) on ice. Protein concentrations were measured using a BCA Protein Assay Kit (23225; Thermo). Equal amounts of total cell extracts were loaded on NuPAGE gels (4–12%, NP0321; Thermo). The gels were run at consistent current (50 mA) for 2 hr at room temperature and then transferred to nitrocellulose blotting membranes (10600016; Life Sciences) at 20 V at 4°C overnight using the NuPAGE system. The membranes were first blocked with 5% milk (70166; Sigma) for 30 min at room temperature, and then incubated with Ty1 (C15200054, 1:1,000 diluted in 5% milk; Diagenode) and histone 3 (ab1791, 1:1,000 diluted in 5% milk; Abcam) antibodies for 2 hr. After five times (5 min each) washing with phosphate‐buffered saline (PBS) plus Tween 20 (27434‐8, 1:1,000; Sigma), membranes were probed with Donkey anti‐Rabbit 800CW and Donkey antimouse 800CW secondary antibodies (926‐32213 and 926‐32212, 1:5,000 in 5% milk; LI‐COR) for 1 hr at room temperature. After five times washing with PBS plus Tween 20, the membranes were analyzed on Odyssey (Westburg).

For the luciferase transfection in both wild‐type and homozygous *Zbtb24* mutant mESCs, 0.5 μg of pGL3_Renilla luciferase and 0.5 μg of pGL3_Firefly luciferase driven by *Cdca7* promoter vectors were transfected. Renilla luciferase was used as a control for balancing the variabilities between the transfections. To restore wild‐type ZBTB24 or ZBTB24 mutant proteins in mutant cells, 1 μg of Ty1_*ZBTB24* or with different missense variants was cotransfected with the two luciferase vectors in homozygous *Zbtb24* mutant mESCs separately. In parallel, the same amount of GFP vector was used in wild‐type and homozygous *Zbtb24* mutant mESCs as a transfection control of overexpression of Ty1_ZBTB24 mutant proteins. Lipofectamine 3000 (L3000008; Thermo) was used as 1:2 ratio following the online protocol. Briefly, 4 µl of lipofectamine was added to 125 µl Knockout DMEM (in terms of 12‐well plate), and 2 µg of total plasmids (two luciferase vectors and the overexpression vector) were mixed with 4 µl of P3000 in 125 µl Knockout DMEM. Then the diluted lipofectamine and plasmid were mixed and incubated at room temperature for 15 min. After incubation, the transfection was done in suspension by combining the mix with 200,000 cells in 250 µl culture medium, and seeding the cells into each well with 500 µl culture medium from a 12‐well plate. After 7 hr, the medium was refreshed using 1 ml normal culture medium. For the transfection in U2OS cells, about four million cells were seeded in one P10 dish. The next day before transfection, the medium was refreshed using 7.5 ml of culture medium without Pen‐Strep. Ty1_*ZBTB24* plasmids with different missense variants per dish were used for transfection with Polyethylenimine (PEI) (23966; Polysciences) reagent in a 1:3 ratio of plasmid and PEI at the concentration of 1 mg/ml (pH 7.4). Briefly, for each dish, 18 µg plasmid was diluted in 1.25 ml DMEM medium, and 54 µl of PEI was added into 1.25 ml DMEM and incubated at room temperature for 5 min. Then, the diluted plasmid and PEI reagent were mixed well and incubated at room temperature for at least 15 min. After incubation, drops of plasmid‐PEI mix were added and the cells were incubated at 37°C overnight. The next day, medium was refreshed with 10 ml of culture medium. Cells were harvested 2 days after transfection for chromatin.

mESCs were harvested 2 days after transfection and treated with the Passive Lysis Buffer from Dual‐Luciferase® Reporter Assay Kit (E1910; Promega). The Luciferase Assay Reagent II and the Stop & Glo® Reagent were prepared following the protocol from the kit. Both renilla and firefly luciferase activities were measured using a Perkin Elmer precisely 1420 Multilabel counter victor 3. The normalized relative activities were calculated by first normalizing to renilla luciferase activity, and then comparing to the normalized activity of wild‐type ZBTB24 overexpression in homozygous *Zbtb24* mutant mESCs.

Chromatin immunoprecipitation (ChIP) was described before (Wu et al., 2016). Briefly, cells were cross‐linked with 1% formaldehyde (344198; Calbiochem) for 10 min at RT and glycine (125 mM) was used to quench cross‐linking for 5 min. Cells were washed twice with cold PBS plus PMSF (93482‐50ML‐F; Sigma) and lysed with NP Buffer (150 mM NaCl, 50 mM Tris–HCl [pH 7.5], 5 mM EDTA, 0.5% NP40, 1% Triton X‐100, protease inhibitor cocktail [05056489001; Roche]). Nuclei were sheared by sonication (Diagenode Bioruptor Pico) with 15 cycles (30 s on/off per cycle). After the sonication, cell debris was pelleted at 10,000 rpm for 5 min at 4°C. Fifty microlitres of the supernatant containing the chromatin was used for DNA isolation with phenol‐chloroform‐isoamylalcohol (15593049; Fisher Scientific). The concentration was measured with Nanodrop and 1 µg isolated DNA was run on a 1.5% agarose gel. A good sonication can be determined by observation of majority of DNA fragments presenting between 300 and 700 base pairs. For the ChIP, protein A and G beads (10002D, 10003D; Life Technologies) were first blocked with PBS plus BSA (A7906, 5 mg/ml; Sigma) and then incubated with 2 µg Ty1 antibody (C15200054; Diagenode) at 4°C for at least 4 hr. Ty1 antibody coupled with beads were then incubated with 30 µg sheared chromatin at 4°C overnight. After immunoprecipitation, beads were washed one time with low‐salt washing buffer (0.1% SDS, 1% Triton X‐100, 2 mM EDTA, 20 mM Tris–HCl [pH 8.1], 150 mM NaCl), high‐salt washing buffer (0.1% SDS, 1% Triton X‐100, 2 mM EDTA, 20 mM Tris–HCl [pH 8.1], 500 mM NaCl), LiCl washing buffer (0.25 M LiCl, 1% NP40, 1% deoxycholate, 1 mM EDTA, 10 mM Tris–HCl [pH 8.1]) and 2 times with TE buffer (10 mM Tris–HCl [pH 8.0], 1 mM EDTA; 10 min incubation at 4°C per time). Input DNA samples were extracted with phenol‐chloroform‐isoamylalcohol DNA after immunoprecipitation was extracted with 10% Chelex (1421253; Bio‐Rad) and used for qPCR analysis. Relative normalized enrichment was calculated by first calculating percentage of input (% input), and then comparing the % input of ZBTB24 mutants to that of wild‐type ZBTB24. Sequences of the primers used for qPCR are shown in Table S1.

Our previous work identified a direct link between *ZBTB24* and the ICF3 gene *CDCA7*. CDCA7 is a zinc finger protein and pathogenic missense variants in this factor cause about 10% of ICF cases (Thijssen et al., 2015). Specifically, we showed that ZBTB24 is a transcription factor that positively regulates CDCA7 expression by binding to and activating the *CDCA7* promoter (Wu et al., 2016). This observation has subsequently been validated in different studies using additional cell lines (Rajshekar et al., 2018; Thompson et al., 2018), demonstrating that this regulation is highly conserved. To dissect how missense variants of *ZBTB24* affect its biological function, we established two independent experimental assays that used *Cdca7* promoter activity and ZBTB24 binding to the *CDCA7* promoter as a readout. First, we used a luciferase reporter assay in mESCs to determine ZBTB24 function on activating the *Cdca7* promoter. Firefly luciferase driven by the *Cdca7* promoter was measured to assess the activity of the *Cdca7* promoter, and renilla luciferase was used as a transfection control. Of note, the lack of a proper chromatin structure, which may influence promoter accessibility and activity, is a limitation of a plasmid‐based reporter strategy. Therefore, in a second assay, we performed ChIP followed by quantitative PCR (qPCR) in a human osteosarcoma cell line (U2OS) to assess ZBTB24 binding to the endogenous *CDCA7* promoter. Since there is currently no ChIP‐suitable ZBTB24 antibody, we used a Ty1‐based overexpression strategy in U2OS cells, which allows high transfection and pulldown efficiencies. We included both ICF2 missense variants (c.1148G>A; p.(Cys383Tyr) and c.1222T>G; p.(Cys408Gly), shown in figure as C383Y and C408G), and the two VUSs identified in the CAKUT and UDN patients (c.1457G>A; p.(Gly486Asp) and c.452C>G; p.(Ser151Cys), shown in Figure 1b as G486D and S151C). In addition, we included gnomAD database variants with different allele frequencies. This allowed us to evaluate the functional consequences of VUSs from healthy individuals. The six gnomAD variants included three rare variants (c.485G>A; p.(Arg162Gln), c.1272T>A; p.(His424Gln) and c.1438C>T; p.(His480Tyr), shown in Figure 1b as R162Q, H424Q, and H480Y), and three relatively high frequency variants (c.146G>A; p.(Arg49Gln), c.1552G>A; p.(Ala518Thr) and c.1672G>A; p.(Asp558Asn), shown in Figure 1b as R49Q, A518T and D558N). While we expect that relatively high‐frequency variants are likely to be benign, low‐frequency variants could be pathogenic (Kobayashi et al., 2017; Richards et al., 2015). Interestingly, for the R49Q variant, there is an entry in the ClinVar database with conflicting interpretations of pathogenicity (Landrum et al., 2016). Background on the tested *ZBTB24* variants is available at the Leiden Open Variation Database (https://databases.lovd.nl/shared/variants/ZBTB24?search_var_status=%3D%22Marked%22%7C%3D%22Public%22).

Stable protein is necessary for proper in vivo function (Stefl, Nishi, Petukh, Panchenko, & Alexov, 2013; Zhang, Miteva, Wang, & Alexov, 2012). Furthermore, about 80% of disease‐associated missense variants were predicted to affect protein stability (Wang & Moult, 2001). Thus, we decided to first assess whether the chosen *ZBTB24* missense variants influence protein stability. We separately introduced each variant into a vector containing Ty1‐tagged full‐length human *ZBTB24* and performed western blot analysis in U2OS cells. Overall, we did not detect any significant differences between wild‐type and missense‐variant containing Ty1_ZBTB24 overexpression constructs (Figure 2a). This data suggests that the tested *ZBTB24* missense variants do not lead to protein instability. Next, we cotransfected a firefly luciferase reporter vector containing the mouse *Cdca7* promoter, a renilla luciferase vector, as well as GFP‐only vector into wild‐type and *Zbtb24* homozygous mutant mESCs (Wu et al., 2016) and measured luciferase activity. As expected, luciferase activity was detected in wild‐type but not *Zbtb24* homozygous mutant mESCs (Figure 2b; Wu et al., 2016). In addition, overexpression of full‐length human Ty1_ZBTB24 could fully restore luciferase activity in *Zbtb24* homozygous mutant mESCs. In contrast, we did not see any luciferase activity when we overexpressed Ty1_ZBTB24 carrying the ICF2 missense variants (C383Y and C408G), consistent with them being pathogenic. The Ty1_ZBTB24 construct carrying the CAKUT variant (G486D) was able to fully restore luciferase activity (Figure 2b), indicating that it does not impair ZBTB24 function in this assay. It has been reported that each ZF domain forms two β‐sheets and one α‐helix, and this structure binds a single zinc ion to stabilize the fold (Wolfe et al., 2000). The zinc ion is tetrahedrally coordinated between two cysteines and histidines to form a compact ββα domain, which is crucial for DNA binding (Wolfe et al., 2000). While all three variants tested are located in the C2H2‐ZF domain, the ICF2 variants are in the two highly conserved cysteine and histidine residues and very likely to destroy the interaction between zinc finger and zinc ion, which will further impact the DNA binding. The CAKUT variant, which is located between the 7th and 8th zinc finger may have little effect on the stability of zinc finger structure. Similarly, the UDN variant, which is located between the BTB and AT‐hook domains (S151C) did not show any impact on *Cdca7* promoter activation, indicating this variant might be nonpathogenic (Figure 2b).

**Figure 2:**
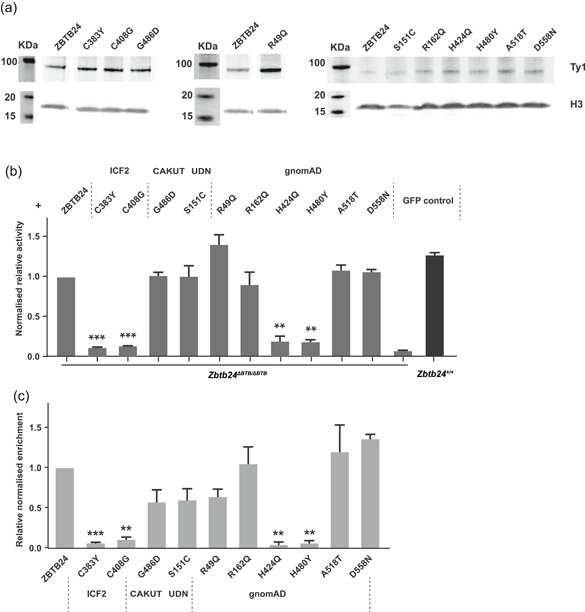
Effects of *ZBTB24* variants on ZBTB24 function. (a) Western blot showing the expression levels of Ty1‐tagged wild‐type ZBTB24 and ZBTB24 with different variants in U2OS cells. H3 is used as a loading control. (b) Luciferase reporter assay showing the relative activity of *Cdca7* promoter (firefly luciferase normalized to renilla luciferase) regulated by wild‐type mouse Zbtb24 and overexpressed human ZBTB24 or with different variants in homozygous *Zbtb24* mutant mESCs. Wild‐type and homozygous *Zbtb24* mutant mESCs transfected with GFP were used as positive and negative control. Error bars = *SEM* from two biological replicates. *t* test ^**^ *p* < .01, ^***^ *p* < .001. (c) ChIP‐qPCR result shows the binding capacities of ZBTB24 or its different variants at *CDCA7* promoter region. Error bars = *SEM* from two independent experiments. *t* test ^**^ *p* < .01, ^***^ *p* < .001. CAKUT, congenital anomalies of the kidneys and urinary tract; ChIP, chromatin immunoprecipitation; gnomAD, Genome Aggregation Database; ICF, immunodeficiency, centromeric instability, facial anomalies; qPCR, quantitative polymerase chain reaction; SEM, standard error of the mean

In the second assay, we assessed the binding ability of ZBTB24 to the *CDCA7* promoter in U2OS cells. ChIP‐qPCR was performed and enrichment of Ty1‐tagged full‐length ZBTB24 or Ty1_ZBTB24 containing our missense variants of interest at the endogenous *CDCA7* promoter was examined. Similar to the luciferase reporter result, the ICF2 variants showed decreased enrichment at the *CDCA7* promoter when compared to wild‐type, whereas the CAKUT and UDN variants did not affect ZBTB24 binding ability (Figure 2c). Therefore, we consider it most likely that the two VUSs are neutral. Nevertheless, while our results demonstrate with high probability that these variants cannot cause ICF, we cannot exclude that they contribute to the observed phenotypes through different mechanisms, which is a limitation of any functional test.

Finally, we tested the six selected gnomAD variants and found that they have distinct effects on ZBTB24 function. The three relatively high frequency variants (R49Q, A518T, and D558N) and the rare variant located in the AT‐hook domain (R162Q), were able to reactivate the *Cdca7* promoter in homozygous *Zbtb24* mutant mESCs (Figure 2b). In addition, ChIP‐qPCR showed enrichment of Ty1_ZBTB24 carrying either of the missense variants at the *CDCA7* promoter (Figure 2c). Together, these results indicate that these variants do not have any impact on ZBTB24 function in our assays. Thus, our results support the classification of c.146G>A (R49Q) as a neutral variant, consistent with the findings from two out of three laboratories in ClinVar. In contrast, luciferase activity and ZBTB24 enrichment were not detected for the other two rare variants (H424Q and H480Y) that are located in the C2H2‐ZF domain (Fig. 2B‐C), demonstrating that they can impair ZBTB24 function. Similar to the two ICF2 variants, the two rare variants (H424Q and H480Y) alter the Histidines in the 5th and 7th C2H2‐ZF motifs, which are, together with the two Cysteines, the key amino acids for C2H2‐ZF formation. Therefore, our data suggest that these individuals in gnomAD are carriers of ICF syndrome.

In conclusion, this study describes two robust functional assays to assess the biological consequences of missense variants in *ZBTB24*. Our system can clearly separate known pathogenic from likely neutral variants, and we show that two VUSs reported in the literature are most likely neutral variants. This type of functional analysis will become increasingly relevant with the wider application of genome‐wide sequencing techniques in clinical diagnostics and underlines the importance of unraveling the pathophysiological mechanisms.

## CONFLICT OF INTERESTS

The authors declare that there is no conflict of interests.

## AUTHOR CONTRIBUTIONS

H.W. contributed to study design, performed experiments, interpreted data and drafted the manuscript. K.K.D.V. performed experiments and interpreted data. S.M.vd.M and G.W.E.S. contributed to study design, interpreted data, and contributed to manuscript writing. L.D. designed and supervised the study, interpreted data, and wrote the manuscript.

## Supporting information

Supplementary information

## References

[humu23786-bib-0001] Bonchuk, A. , Denisov, S. , Georgiev, P. , & Maksimenko, O. (2011). Drosophila BTB/POZ domains of "ttk group" can form multimers and selectively interact with each other. Journal of Molecular Biology, 412(3), 423–436. 10.1016/j.jmb.2011.07.052 21821048

[humu23786-bib-0002] van den Boogaard, M. L. , Thijssen, P. E. , Aytekin, C. , Licciardi, F. , Kiykim, A. A. , Spossito, L. , … van der Maarel, S. M. (2017). Expanding the mutation spectrum in ICF syndrome: Evidence for a gender bias in ICF2. Clinical Genetics, 92(4), 380–387. 10.1111/cge.12979 28128455

[humu23786-bib-0003] Cerbone, M. , Wang, J. , Van der Maarel, S. M. , D'Amico, A. , D'Agostino, A. , Romano, A. , & Brunetti‐Pierri, N. (2012). Immunodeficiency, centromeric instability, facial anomalies (ICF) syndrome, due to ZBTB24 mutations, presenting with large cerebral cyst. American Journal of Medical Genetics. Part A, 158A(8), 2043–2046. 10.1002/ajmg.a.35486 22786748 PMC3402683

[humu23786-bib-0004] Chouery, E. , Abou‐Ghoch, J. , Corbani, S. , El Ali, N. , Korban, R. , Salem, N. , … Megarbane, A. (2012). A novel deletion in ZBTB24 in a Lebanese family with immunodeficiency, centromeric instability, and facial anomalies syndrome type 2. Clinical Genetics, 82(5), 489–493. 10.1111/j.1399-0004.2011.01783.x 21906047

[humu23786-bib-0005] Conrad, M. A. , Dawany, N. , Sullivan, K. E. , Devoto, M. , & Kelsen, J. R. (2017). Novel ZBTB24 mutation associated with immunodeficiency, centromere instability, and facial anomalies type‐2 syndrome identified in a patient with very early onset inflammatory bowel disease. Inflammatory Bowel Diseases, 23(12), 2252–2255. 10.1097/MIB.0000000000001280 29023266 PMC5685903

[humu23786-bib-0006] Abecasis, G. R. , Altshuler, D. , Auton, A. , Brooks, L. D. , Durbin, R. M. , Gibbs, R. A. , … McVean, G. A. (2010). A map of human genome variation from population‐scale sequencing. Nature, 467(7319), 1061–1073. 10.1038/nature09534 20981092 PMC3042601

[humu23786-bib-0007] Gonzaga‐Jauregui, C. , Lupski, J. R. , & Gibbs, R. A. (2012). Human genome sequencing in health and disease. Annual Review of Medicine, 63, 35–61. 10.1146/annurev-med-051010-162644 PMC3656720 22248320

[humu23786-bib-0008] de Greef, J. C. , Wang, J. , Balog, J. , den Dunnen, J. T. , Frants, R. R. , Straasheijm, K. R. , & van der Maarel, S. M. (2011). Mutations in ZBTB24 are associated with immunodeficiency, centromeric instability, and facial anomalies syndrome type 2. American Journal of Human Genetics, 88(6), 796–804. 10.1016/j.ajhg.2011.04.018 21596365 PMC3113345

[humu23786-bib-0009] Kamae, C. , Imai, K. , Kato, T. , Okano, T. , Honma, K. , Nakagawa, N. , … Nonoyama, S. (2018). Clinical and immunological characterization of ICF syndrome in Japan. Journal of Clinical Immunology, 38(8), 927–937. 10.1007/s10875-018-0559-y 30353301

[humu23786-bib-0010] Kobayashi, Y. , Yang, S. , Nykamp, K. , Garcia, J. , Lincoln, S. E. , & Topper, S. E. (2017). Pathogenic variant burden in the ExAC database: An empirical approach to evaluating population data for clinical variant interpretation. Genome Medicine, 9(1), 13 10.1186/s13073-017-0403-7 28166811 PMC5295186

[humu23786-bib-0011] Landrum, M. J. , Lee, J. M. , Benson, M. , Brown, G. , Chao, C. , Chitipiralla, S. , … Maglott, D. R. (2016). ClinVar: Public archive of interpretations of clinically relevant variants. Nucleic Acids Research, 44(D1), D862–D868. 10.1093/nar/gkv1222 26582918 PMC4702865

[humu23786-bib-0012] Lek, M. , Karczewski, K. J. , Minikel, E. V. , Samocha, K. E. , Banks, E. , Fennell, T. , … MacArthur, D. G. (2016). Analysis of protein‐coding genetic variation in 60,706 humans. Nature, 536(7616), 285–291. 10.1038/nature19057 27535533 PMC5018207

[humu23786-bib-0013] Lyst, M. J. , Connelly, J. , Merusi, C. , & Bird, A. (2016). Sequence‐specific DNA binding by AT‐hook motifs in MeCP2. FEBS Letters, 590(17), 2927–2933. 10.1002/1873-3468.12328 27461740 PMC5028900

[humu23786-bib-0014] Najafabadi, H. S. , Mnaimneh, S. , Schmitges, F. W. , Garton, M. , Lam, K. N. , Yang, A. , … Hughes, T. R. (2015). C2H2 zinc finger proteins greatly expand the human regulatory lexicon. Nature Biotechnology, 33(5), 555–562. 10.1038/nbt.3128 25690854

[humu23786-bib-0015] Nitta, H. , Unoki, M. , Ichiyanagi, K. , Kosho, T. , Shigemura, T. , Takahashi, H. , & Sasaki, H. (2013). Three novel ZBTB24 mutations identified in Japanese and Cape Verdean type 2 ICF syndrome patients. Journal of Human Genetics, 58(7), 455–460. 10.1038/jhg.2013.56 23739126

[humu23786-bib-0016] Peterson, T. A. , Doughty, E. , & Kann, M. G. (2013). Towards precision medicine: Advances in computational approaches for the analysis of human variants. Journal of Molecular Biology, 425, 4047–4063. Nov 1 23962656 10.1016/j.jmb.2013.08.008 PMC3807015

[humu23786-bib-0017] Rajshekar, S. , Yao, J. , Arnold, P. K. , Payne, S. G. , Zhang, Y. , Bowman, T. V. , … Goll, M. (2018). Pericentromeric hypomethylation elicits an interferon response in an animal model of ICF syndrome. eLife, 7, e39658 10.7554/eLife.39658 30484769 PMC6261255

[humu23786-bib-0018] Richards, S. , Aziz, N. , Bale, S. , Bick, D. , Das, S. , Gastier‐Foster, J. , … Committee, A. L. Q. A. (2015). Standards and guidelines for the interpretation of sequence variants: A joint consensus recommendation of the American College of Medical Genetics and Genomics and the Association for Molecular Pathology. Genetics in Medicine, 17(5), 405–424. 10.1038/gim.2015.30 25741868 PMC4544753

[humu23786-bib-0019] Stefl, S. , Nishi, H. , Petukh, M. , Panchenko, A. R. , & Alexov, E. (2013). Molecular mechanisms of disease‐causing missense mutations. J Mol Biol, 425(21), 3919–3936. 10.1016/j.jmb.2013.07.014 23871686 PMC3796015

[humu23786-bib-0020] Stenson, P. D. , Ball, E. V. , Mort, M. , Phillips, A. D. , Shiel, J. A. , Thomas, N. S. , … Cooper, D. N. (2003). Human Gene Mutation Database (HGMD): 2003 update. Human Mutatations, 21(6), 577–581. 10.1002/humu.10212 12754702

[humu23786-bib-0021] Stogios, P. J. , Downs, G. S. , Jauhal, J. J. , Nandra, S. K. , & Prive, G. G. (2005). Sequence and structural analysis of BTB domain proteins. Genome Biology, 6(10), R82 10.1186/gb-2005-6-10-r82 16207353 PMC1257465

[humu23786-bib-0022] Thijssen, P. E. , Ito, Y. , Grillo, G. , Wang, J. , Velasco, G. , Nitta, H. , … Sasaki, H. (2015). Mutations in CDCA7 and HELLS cause immunodeficiency‐centromeric instability‐facial anomalies syndrome. Nature Communications, 6, 7870 10.1038/ncomms8870 PMC4519989 26216346

[humu23786-bib-0023] Thompson, J. J. , Kaur, R. , Sosa, C. P. , Lee, J. H. , Kashiwagi, K. , Zhou, D. , & Robertson, K. D. (2018). ZBTB24 is a transcriptional regulator that coordinates with DNMT3B to control DNA methylation. Nucleic Acids Research, 46(19), 10034–10051. 10.1093/nar/gky682 30085123 PMC6212772

[humu23786-bib-0024] Vivante, A. , Hwang, D. Y. , Kohl, S. , Chen, J. , Shril, S. , Schulz, J. , … Hildebrandt, F. (2017). Exome sequencing discerns syndromes in patients from consanguineous families with congenital anomalies of the kidneys and urinary tract. Journal of the American Society of Nephrology, 28(1), 69–75. 10.1681/ASN.2015080962 27151922 PMC5198271

[humu23786-bib-0025] Wang, Z. , & Moult, J. (2001). SNPs, protein structure, and disease. Human Mutations, 17(4), 263–270. 10.1002/humu.22 11295823

[humu23786-bib-0026] Weemaes, C. M. , van Tol, M. J. , Wang, J. , van Ostaijen‐ten Dam, M. M. , van Eggermond, M. C. , Thijssen, P. E. , … van der Maarel, S. M. (2013). Heterogeneous clinical presentation in ICF syndrome: Correlation with underlying gene defects. European Journal of Human Genetics, 21(11), 1219–1225. 10.1038/ejhg.2013.40 23486536 PMC3798845

[humu23786-bib-0027] Wolfe, S. A. , Nekludova, L. , & Pabo, C. O. (2000). DNA recognition by Cys2His2 zinc finger proteins. Annual Review of Biophysics and Biomolecular Structure, 29, 183–212. 10.1146/annurev.biophys.29.1.183 10940247

[humu23786-bib-0028] Woods, N. T. , Baskin, R. , Golubeva, V. , Jhuraney, A. , De‐Gregoriis, G. , Vaclova, T. , … Monteiro, A. N. (2016). Functional assays provide a robust tool for the clinical annotation of genetic variants of uncertain significance. NPJ Genomic Medicine, 1, 16001–16009. 10.1038/npjgenmed.2016.1 28781887 PMC5539989

[humu23786-bib-0029] Wu, H. , Thijssen, P. E. , de Klerk, E. , Vonk, K. K. , Wang, J. , den Hamer, B. , … Daxinger, L. (2016). Converging disease genes in ICF syndrome: ZBTB24 controls expression of CDCA7 in mammals. Human Molecular Genetics, 25(18), 4041–4051. 10.1093/hmg/ddw243 27466202

[humu23786-bib-0030] Xu, G. L. , Bestor, T. H. , Bourc'his, D. , Hsieh, C. L. , Tommerup, N. , Bugge, M. , & Viegas‐Pequignot, E. (1999). Chromosome instability and immunodeficiency syndrome caused by mutations in a DNA methyltransferase gene. Nature, 402(6758), 187–191. 10.1038/46052 10647011

[humu23786-bib-0031] Zhang, Z. , Miteva, M. A. , Wang, L. , & Alexov, E. (2012). Analyzing effects of naturally occurring missense mutations. Computational and Mathematical Methods in Medicine, 2012, 805827–15. 10.1155/2012/805827 22577471 PMC3346971

