# Supplementary material for: A functional assay to classify ZBTB24 missense variants of unknown significance
Source: Hum Mutat. 2019 Jun 18;40(8):1077–83. doi: 10.1002/humu.23786 (PMC6771626; doi:10.1002/humu.23786)
Supplement: Supplementary file 1 — Supplementary information [file HUMU-40-1077-s001.docx]

**Supp. Table S1**

**Supp. Table 1**

List of primers used

| Primers for mutagenesis | Sequence |
| --- | --- |
| ZBTB24_1148G>A_Fw | CCTGTGATCAATACGGAAAATATTTC |
| ZBTB24_1148G>A_Rev | GAAATATTTTCCGTATTGATCACAGG |
| ZBTB24_1222T>G_Fw | CACTCATTACCGGAAGGCAAAGACTGCCATC |
| ZBTB24_1222T>G_Rev | GATGGCAGTCTTTGCCTTCCGGTAATGAGTG |
| ZBTB24_1457G>A_Fw | GCATTCTACACACTGACAAGAAGCCTTTCTC |
| ZBTB24_1457G>A_Rev | GAGAAAGGCTTCTTGTCAGTGTGTAGAATGC |
| ZBTB24_452C>G_Fw | CCCCAGTGGTTGTTATCTGTAATAAGAAAAACGATCC |
| ZBTB24_452C>G_Rev | GGATCGTTTTTCTTATTACAGATAACAACCACTGGGG |
| ZBTB24_146G>A_Fw | GAGAATGTACATTTCCAGGCCCACAAAGCCTTAC |
| ZBTB24_146G>A_Rev | GTAAGGCTTTGTGGGCCTGGAAATGTACATTCTC |
| ZBTB24_485G>A_Fw | CCTCCAAAGCGGAAACAGGGAAGACCAAAAAAAG |
| ZBTB24_485G>A_Rev | CTTTTTTTGGTCTTCCCTGTTTCCGCTTTGGAGG |
| ZBTB24_1272T>A_Fw | CTCAGCTAAAGAAACAACTGCGAACACACACAG |
| ZBTB24_1272T>A_Rev | CTGTGTGTGTTCGCAGTTGTTTCTTTAGCTGAG |
| ZBTB24_1438C>T_Fw | CAGTGCCAAAAGGAGATACTGCATTCTACACAC |
| ZBTB24_1438C>T_Rev | GTGTGTAGAATGCAGTATCTCCTTTTGGCACTG |
| ZBTB24_1552G>A_Fw | GCAAGGAGAAGCATACTTCAGATGCCAGC |
| ZBTB24_1552G>A_Rev | GCTGGCATCTGAAGTATGCTTCTCCTTGC |
| ZBTB24_1672G>A_Fw | GCTTCTCGTAACCAATTCTGTACATAAC |
| ZBTB24_1672G>A_Rev | GTTATGTACAGAATTGGTTACGAGAAGC |
|  |  |
| Primers for ChIP-qPCR | Sequence |
| CDCA7_pro_Fw | GTCCCCGTAGCCCCTAAAG |
| CDCA7_pro_Rev | GTCCTGCAAGCTGTGGTAAC |
